# Effects of Low-Frequency Pulsed Electromagnetic Fields on High-Altitude Stress Ulcer Healing in Rats

**DOI:** 10.1155/2019/6354054

**Published:** 2019-06-11

**Authors:** Mingke Jiao, Hong Yin, Jie Hu, Wenjuan Xu, Xiao Zhang, Peng Zhang

**Affiliations:** ^1^The Departments of Radiology, Xijing Hospital, Fourth Military Medical University, Xi'an 710032, China; ^2^The Department of Medical Engineering, General Hospital of Xinjiang Military Region, Urumqi 830000, China; ^3^The Department of Echocardiography, Affiliated Traditional Chinese Medicine Hospital, Xinjiang Medical University, Urumqi 830000, China

## Abstract

High-altitude stress ulcer (HSU) has constantly been a formidable clinical challenge for high-altitude and severe hypoxia. Pulsed electromagnetic fields (PEMFs) have been verified to have the ability to penetrate tissues, and the biological effects have been confirmed effective on various tissue restorations. However, the therapeutic effect of PEMFs on HSU has been rarely reported. This study aimed to evaluate the effects of PEMFs on HSU healing systematically. Sprague–Dawley rats were assigned to control, HSU, and HSU+PEMF groups. The HSU models were induced by restraint stress under low-pressure hypoxia. The HSU+PEMF group was subjected to PEMF exposure. During the HSU healing, gastric juice pH values, ulcer index (UI), and histopathologic changes were investigated. Furthermore, tumor necrosis factor-*α* (TNF-*α*) was determined to analyze the severity of gastric membrane inflammations. Norepinephrine (NE), which influences gastric acid secretion, was measured. Results indicated the UI of the HSU+PEMF decreased faster than that of the HSU group. Histopathologic observation suggested that the ulcer tissue healing is faster in the HSU+PEMF group than in the HSU group. The TNF-*α*/total protein results revealed that the inflammation of the HSU+PEMF group is suppressed effectively. The pH values are higher in the HSU+PEMF group than in the HSU, as confirmed by NE examination. The results indicated that low-frequency PEMFs can penetrate stomach tissues to relieve the symptoms of HSU and promote the regeneration of disturbed tissues, thus implying the clinical potential of PEMF therapy for HSU treatment.

## 1. Introduction

Stress ulcer mainly occurs in high-altitude and plateau regions due to severe hypoxia, colds, and other stresses [1]. An ulcer is accompanied by bleeding and infiltration of inflammatory cells into the gastric mucosa [2]. HSU in high-altitude and cold regions may result in heavy bleeding and perforation of the digestive tract, even death without timely and effectively treatment. As the main factors associated with the induction of HSU, cold and severe hypoxia increases the blood viscosity of gastric mucosa, thereby resulting in thrombosis and ulcer [3, 4]. In addition, the environment stress, which induced the increased central nervous system activity, may increase gastric acid secretion and causes spasm on the small vessels of mucosa [5, 6]. Currently, drugs and surgical resection remain the main therapy for gastric ulcers. For example, certain drugs have been used to influence the gastric acid secretion and norepinephrine (NE). However, several side effects of drugs and surgery have caused injuries to patients, especially in high-altitude regions; these injuries include mental aberrations, asiderosis, and pneumonia [7–9]. Thus far, few methods have successfully treated HSU. Therefore, HSU remains a clinical challenge given complicated plateau stress caused by a combination of colds, hypoxia, and so on. Therefore, effective and low-cost therapies must be developed to accelerate HSU healing.

Substantial and growing evidence has confirmed that low-frequency pulsed electromagnetic fields (PEMFs), as an alternative noninvasive method, can efficiently induce tissue regeneration and cell proliferation [10–12]. Therefore, the positive effects of PEMFs on the healing of certain tissue damage have been investigated in numerous animal experiments [13, 14]. In addition, clinical investigations have confirmed the efficacy of PEMFs in repairing impaired tissues [4, 15]. Moreover, the penetration characteristic of PEMFs has been applied to treat injured tissues in deep layers [16]. Specifically, several experimental studies have demonstrated that PEMFs can adjust the NE level to minimize the psychological stresses of biology [1, 17]. HSU differs from other tissue injuries in many aspects. First, the cause of HSU is related to stresses [5], which may be associated with NE. Second, the injured tissues of HSU are found inside the body. Finally, HSU pathology is characterized by small vessel blockage and cell apoptosis. Therefore, to date, there have been no studies investigating the effectiveness of low-frequency PEMF exposure in accelerating HSU healing.

In the present study, we systematically determined the noninvasive low-frequency PEMFs on HSU in rats in terms of stress-induced gastric ulcer development or healing of tissue lesions. According to the positions of ulcer and previous studies, 15 Hz and 34 ± 0.4 mT of PEMFs were applied to effectively induce biological effects and tissue healing. Accordingly, NE was measured to determine the influence of PEMFs on central nervous system activity. In addition, gastric juice pH values, ulcer index (UI), associated protein of inflammation, and histological peculiarity of different groups were systematically determined and compared during HSU wound healing. Ultimately, the purpose of the research was to develop an appropriately characterized noninvasive treatment for HSU via low-frequency PEMFs.

## 2. Materials and Methods

### 2.1. Preparation of Animals

In this experiment, 72 Sprague–Dawley rats (220 ± 20 g) of either sex were purchased from the Animal Center of the Fourth Military Medical University. The study was performed strictly in accordance with the guiding principles of the Institutional Animal Ethical Committee, the Committee for the Purpose of Control and Supervision of Experiments on Animals, and the Guide for the Care and Use of Laboratory Animals published by the National Institute of Health (NIH Publication No. 85-23). The animals received standard food and clean tap water ad libitum from an automated watering system and were kept at room temperature (24 ± 1°C) before HSU model construction.

### 2.2. HSU Models

The rats were assigned to three groups, namely, normal control (n = 8), HSU (n = 32), and HSU with low-frequency PEMF exposure (HSU+PEMF, n = 32). First, the rats from the HSU and HSU+PEMF groups were housed for 2 h a day in a large hypobaric chamber (DYC-3013M, Urumqi General Hospital of Lanzhou Military Region, Urumqi, China) with controlled temperature (24 ± 1°C), humidity (50%  ± 10%), and pressure (54.1 ± 1.0 kPa) for 5 days to acclimatize them under hypoxic and low-pressure conditions. For the stomach to be involved in stress responses, during the acclimatization course, the rats were fasted, bounded, and immersed carefully in 10°C water with surface and breastbone line for half an hour per day. On the fifth day of the acclimatization, the rat stomachs were further perfused with 2 ml/kg pure alcohol to ensure a perfect HSU model. After model establishment, the rats from the HSU+PEMF group were exposed to low-frequency PEMFs for 3 h daily at the normal comfortable environment (24 ± 1°C) for 8 days. During the PEMF exposure, the rats were housed in transparent resin cages (60mm × 120 mm × 55 mm) and diminished range of motion to ensure that the PEMF could focus on the stomach. During the HSU healing, all of the groups were individually housed and fed at a normal temperature (24 ± 1°C) and pressure (95.3±1.3 kPa). At a 2-day interval during the HSU healing, the UI, juice pH values, histological changes, tumor necrosis factor-*α* (TNF-*α*)/total protein, and NE of eight rats from each group were examined and evaluated.

### 2.3. PEMF Exposures

**Figure 1:**
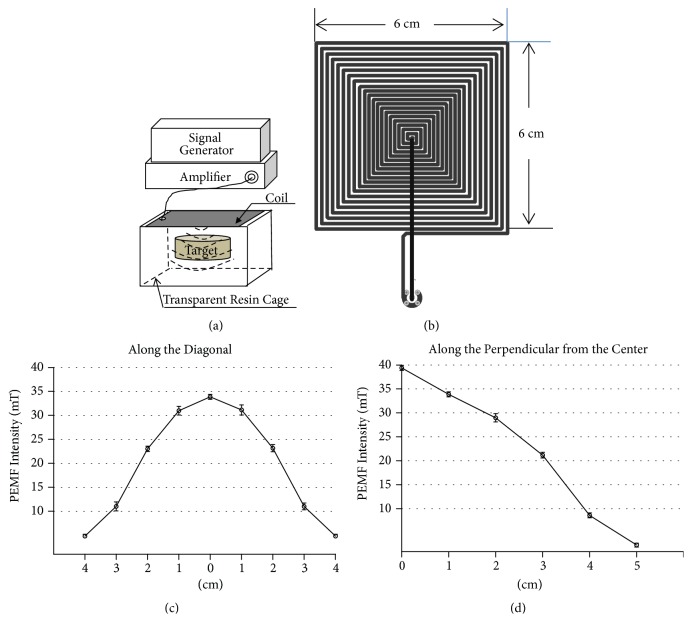
(a) Schematic of stimulators used to expose rats to PEMF. (b) Diagram of the focused coil. (c) PEMF spatial distribution along diagonal. (d) PEMF spatial distribution along perpendicular from the center.

During the ulcer healing, the rats from the HSU+PEMF group were subjected to a whole-body PEMF exposure for 7 days. The low-frequency PEMF facility (BPS-I, Urumqi, China; China Patent no. 201520046119.2) consisted of an electromagnetic generator and a transparent resin cage with focused coil (Figure 1(a)). The low-frequency pulsed signals were produced by the signal generator, which was designed with the chip-microcomputer (STC89C52, STC, Shenzhen, China). The frequency and duty cycle were adjusted and controlled with the specific program. And then the output signal amplitude was amplified by the power amplifier. The cages were covered with square focus coils, which provide a top-down electromagnetic field exposure. A transmitted electromagnetic field coil was composed of a printed circuit board with 20 turns of square rolling copper, which has a width of 6 cm (Figure 1(b)). The focused coil ensured that the electromagnetic field can concentrate the main energy to the stomach region of rats by postural restriction. Moreover, the total bodies of rats were influenced by PEMFs. Space electromagnetic field strength distribution along the diagonal at a perpendicular distance of 1 cm to the coil was determined by using a Tesla meter (HT108; Hengtong, Shanghai, China). Meanwhile, the electromagnetic field strength distribution along the perpendicular of the focused coil from the center to 5 cm was measured. The acquired electromagnetic field intensity data were graphically represented (Figures 1(c) and 1(d)). The central point of the electromagnetic fields reached a maximum strength of 34 ± 0.4 mT, and the field strength decreased as the horizontal and perpendicular distance from the central coil increased. During the experiment, electromagnetic fields with 15 Hz, 40% duty cycle, were used. According to the International Commission on Non-Ionizing Radiation Protection (ICNIRP) guidelines, the current PEMF had not the negative biological effects on the organism. The rats were housed in transparent resin cages with air bleed, thereby allowing an approximately 1 cm distance between the coil and rat dorsum. In addition, background electromagnetic fields were measured as 3 ± 0.3 *μ*T in the experiment.

### 2.4. Gastric Juice pH Values and UI

During the HSU healing, on Days 1, 3, 5, and 7 after the model operation, eight rats in each group were slaughtered by using 1.5 ml kg−15% chloral hydrate injection. The abdominal cavity was cut for gastric mobilization down the centerline from the xiphoid process to the lower abdomen. After the cardiac and pyloric were nipped with a hemostat, a pH meter was inserted through a small incision to the stomach to measure the gastric juice pH values. Then, the stomach was opened along the small curvature and was pinned in the saline with mucosae eversion to conduct macroscopic examination and scoring of UI. Lesion size based on erosion and bleeding conditions was measured along its greatest diameter by using a magnifying glass (Tunçel, Tunçel, & Aboul-Enein, 2003). Instruction sheets with a five-point scale (1: lesion size≤1 mm; 2: 1 mm ≤ lesion size≤2 mm; 3: 2 mm ≤ lesion size≤3 mm; 4: 3 mm ≤ lesion size≤4 mm; and 5: 4 mm ≤ lesion size) were prepared for the measurement on each group of rats. Five petechial lesions were considered equivalent to a 1 mm ulcer. The final UI of each group was calculated by dividing the total scores in each group by the number of animals.

### 2.5. Histopathologic Changes

On Days 1, 3, 5, and 7 after the measurements of pH and UI values, 2 mm × 5 mm stomach mucosae samples of eight rats from the HSU and HSU+PEMF groups were harvested for histological examination every time. The ulcer tissue specimens were excised, directly immersed, and fixed in a 10% formalin solution for 8 h. The samples were embedded in paraffin wax, and 5 *μ*m-thick sections were cut by using a saw microtome (Leica RM 2155; Leica Instruments, Nussloch, Germany). The sections were deparaffinized, rehydrated, followed by Mayer's hematoxylin and eosin staining. The slides were observed under a light microscope, and the histopathologic differences between the HSU and HSU+PEMF groups were evaluated.

### 2.6. TNF-*α* Protein Assay

TNF-*α* is closely associated with the inflammation indexes of gastric mucosa [18]; thus, TNF-*α* values were detected here. Appropriate amounts of gastric mucosa of eight rats from different groups were cut and homogenized in 400 ml PBS (pH 7.2). The homogenates were centrifuged at 3,000 rpm for 10 min at 4°C. The supernatant was collected for subsequent analysis. The TNF-*α* concentration was determined by using an enzyme-linked immunosorbent assay kit specific for TNF-*α* (Biosource, Camarillo, CA, USA) according to the manufacturer's specifications.

### 2.7. NE Measurements

NE concentrations were measured by using a high-pressure liquid chromatographic (HPLC) assay combined with electrochemical detection (ECD) (Agilent 100, USA). The hippocampus of rat brains was cryopreserved and smashed with ultrasound. On the basis of an internal standard, 20 ml dihydroxybenzamidine was added to the samples, in which 200 *μ*l (0.1 mol/l) HClO_4_ was added and vortex mixed for 5 min. The resultant homogenates were centrifuged for 10 min. The NE concentrations of the supernatant were measured by HPLC with ECD. During the operation, all the processes were completed by the same person on the basis of the instruction for reagents and instruments.

### 2.8. Statistical Analysis

All data were expressed as mean ± deviation (S.D.). Gastric juice pH values, UIs, histological changes, TNF-*α* protein, and NE concentrations of each group were analyzed in this study. ANOVA was conducted to evaluate the differences among the various groups. Fisher's least significant difference t-test was applied to determine the significant pairwise differences between the groups. All analyses were performed in SPSS (version 10.0) statistical software for Windows (SPSS, Chicago, IL, USA). Values with P < 0.05 were considered statistically significant.

## 3. Results and Discussion

### 3.1. Gastric Juice pH Values and UIs

**Figure 2:**
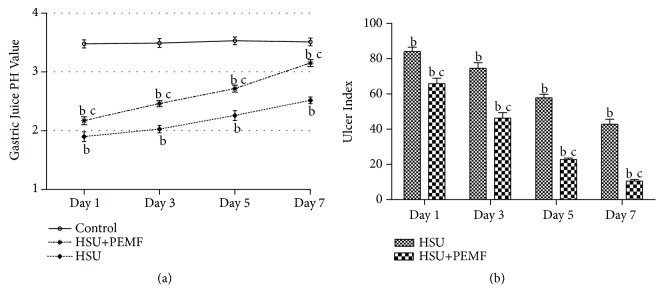
Comparison of the pH and UI values between the HSU and HSU+PEMF groups on Days 1, 3, 5, and 7. The values are expressed as mean ± SD. ^b^P < 0.01 versus the control group and ^c^P < 0.05 versus the HSU group.

Figures 2(a) and 2(b) illustrate the results of average gastric juice pH values and UIs of different groups on Days 1, 3, 5, and 7.  Figure 2(a) demonstrates that the pH of the control group was similar at approximately 3.49±0.6, and the pH values were lower in the HSU and HSU+PEMF groups than in the control group and increase with time (P < 0.01). However, the pH values were higher in the HSU+PEMF group than in the HSU group on Days 3, 5, and 7 (P < 0.05), although no obvious difference was observed on Day 1. In Figure 2(b), the UI difference between the HSU and HSU+PEMF groups was obvious during the ulcer healing. The UI values were higher in the HSU group than in the HSU+PEMF group (P < 0.05). On Day 7, the UI of the HSU+PEMF group reached 10.25±.03, thus indicating that the ulcer is nearly restored.

### 3.2. Histological Changes

**Figure 3:**
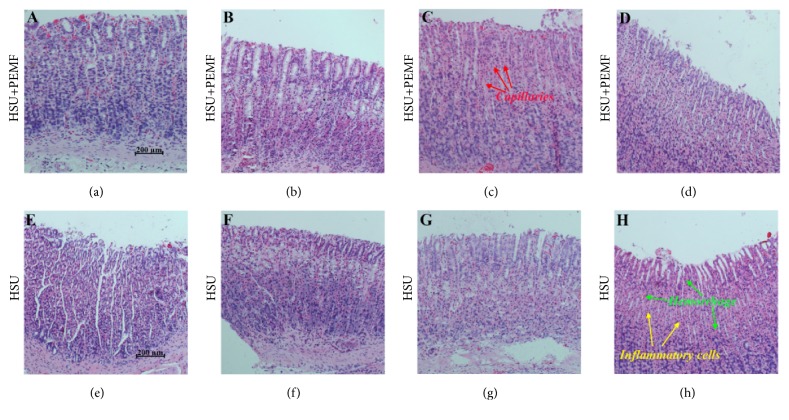
Histology of the HSU+PEMF and HSU wounds in rats (hematoxylin and eosin staining; 10×): (a and e) Day 1, (b and f) Day 3, (c and g) Day 5, and (d and h) Day 7.

Histological examination revealed that no remarkable histological changes occur in the control rats. On Days 1, 3, 5, and 7, the histological changes and differences between the HSU+PEMF and HSU groups were confirmed, as depicted (Figure 3). On Day 1, the normal structure of stomach mucosae nearly disappeared, and considerable edema, inflammatory cell infiltration, and exudative hemorrhage are displayed (Figures 3(a) and 3(e)). However, the degree and incidence of edema, exudative hemorrhage, and inflammatory cell infiltration were remarkably higher in the HSU group than in the HSU+PEMF group. On Days 3 and 5, the structure of stomach mucosae of the two groups accumulated, and the structure was clearer in the HSU+PEMF group than in the HSU group (Figures 3(c) and 3(g)). During restoration, edema, exudative hemorrhage, and inflammatory cell infiltration remarkably decreased and were still lower in the HSU+PEMF group than in the HSU group. Figures 3(b) and 3(c) exhibit that the newly formed capillaries (red arrows) and fibrous connective tissues of the HSU+PEMF group were obtained, but were not observed in the HSU group (Figures 3(f) and 3(g)). On Day 7, edema, exudative hemorrhage, and inflammatory cell infiltration were nearly absent in the HSU+PEMF group, and the mature capillary network and stomach mucosae were nearly formed (Figure 3(d)). However, sparse inflammatory cells (yellow arrows), hemorrhage (green arrows), and spare edema could still be observed in the HSU group, and some hemorrhage could be found even in deep layer (Figure 3(h)). In addition, the structure of stomach mucosae was obscure.

### 3.3. TNF-*α* Protein

**Figure 4:**
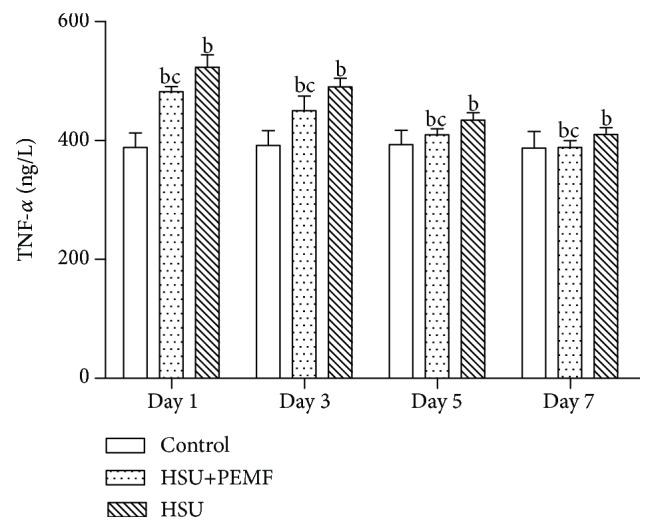
Comparison of TNF-*α* protein between each group on Days 1, 3, 5, and 7. The values are expressed as mean ± SD. ^b^P < 0.01 versus control group. ^c^P < 0.05 versus HSU group.

Figure 4 presents that the TNF-*α* protein of the control group remained at approximately 396±3 ng/L on Days 1, 3, 5, and 7. On Days 1, 3, and 5, the TNF-*α* values were higher in the HSU and HSU+PEMF groups than in the control group (P < 0.01). However, the TNF-*α* values were significantly lower in the HSU+PEMF group than in the HSU group (P < 0.05). However, on Day 7, the TNF-*α* value of the HSU+PEMF group reached approximately 397±4, which has no significant difference with the control group (P < 0.01). Moreover, a remarkable difference between the TNF-*α* value of the HSU and the control groups was observed. In particular, these results indicated that the inflammation of ulcer of the HSU+PEMF group nearly disappears on Day 7.

### 3.4. NE

NE is closely related to nervous irritability and can influence stomach acid through the sympathetic nervous system [19]. In Table 1, the NE concentrations of the rats were lower in the control group than in the other groups (P < 0.01) at different time points. The NE concentrations of the HSU and HSU+PEMF groups decreased with time. Moreover, Table 1 summarizes the significant difference between the NE concentrations of the HSU and HSU+PEMF groups from Day 1 to Day 7. In addition, the NE concentrations were lower in the HSU+PEMF group than in the HSU group at different times (P < 0.05). On Day 7, the NE concentrations of the HSU+PEMF group reached 186.23±9.29, which was nearly close to the values of the control group (P < 0.01). However, the NE concentrations remained significantly higher in the HSU group than in the control group.

## 4. Discussion

In the last decades, numerous studies have reported that PEMFs have multiple effects on living organisms by altering the permeability and ion transfer of cellular membranes [13, 20, 21]. Therefore, PEMFs have been investigated for the treatment of a wide spectrum of diseases, such as skin wounds and bone fractures [16, 22]. However, the effect of low-frequency PEMFs on the wound healing of the inner structure of organisms, such as the stomach, has not been described. In the current study, the effects of low-frequency PEMFs on stomach HSU healing were systematically investigated in HSU rat models. The results indicated that low-frequency PEMFs can penetrate body tissues to remarkably accelerate HSU healing.

In general, the pathophysiology of stomach HSU is different from normal skin wounds. First, the pathological conditions of the HSU come from physical harm and stress influences [5, 23]. In addition, the cure of stomach ulcer depends on the anaerobic environment of an abdominal cavity. On the basis of the abovementioned pathophysiological factors and specificity of PEMFs, PEMFs were used to influence the cure of HSU and were verified to improve the cure of HSU. Similar results were reported in our previous studies, in which PEMFs can penetrate tissues to accelerate the healing of frostbite tissues [16]. Moreover,* Helicobacter pylori* (HP) infection is another factor that can result in HSU [24]. Some works have indicated that the PEMF has bactericidal effect on some bacteria [25, 26]. However, the effect of PEMF on HP remains unknown. Therefore, the effect of PEMF on HP in vitro and in vivo will be explored as another special subject in our further work. In this study, PEMFs with 15 Hz have been used because the penetration depth of electromagnetic fields increases with the decrease in frequency. Consequently, all HSUs could be exposed to low-frequency PEMFs during treatment. In addition, the entire bodies of rats were nearly exposed to PEMFs in the present study although the stomach was located in the central of electromagnetic field energy. The positive effect of the entire exposure style on HSU requires subsequent experiments.

The degree of HSU was closely associated with the pH values of gastric juice [27]. Our observation revealed that the pH values of the HSU models were considered below the normal value. Correspondingly, the UI values for the HSU and HSU+PEMF groups were higher on Day 1 than on the other days. The pH values increased faster in the HSU+PEMF group than in the HSU group with PEMF exposure at different times. Furthermore, the pH value of the HSU+PEMF group on Day 7 nearly reached the control group. Moreover, the UI values were lower in the HSU+PEMF group than in the HSU group at all time-points. Therefore, PEMFs accelerated the decrease in the UI value, and the increases in pH values promoted HSU healing. In addition, the HSU group showed that the level of NE is elevated by stress. Similar results were also reported in several previous studies [19, 28]. However, PEMFs effectively decreased the NE concentrations of rats on the basis of the results of NE concentrations, which control the stomach neurotransmitter to suppress stomach acids by binding to an adrenergic alpha receptor. Therefore, the series of influence of PEMFs on the HSU group could be called a snowball effect. The influence of PEMFs on NE played a crucial role. However, the effect of PEMFs on the total sympathetic nerve remained unknown. The participation of NE or total sympathetic nerve in the course of HSU healing could not be determined. Therefore, the influence of PEMFs on the total sympathetic nerve should be clarified in subsequent studies.

The histological examination indicated that the ulcer cure is obviously faster in the HSU+PEMF group than in the HSU group. During the examination, the inflammatory cell infiltration diminished quicker in the HSU+PEMF group than in the HSU group, and more mature capillary network and fibrous connective tissue were observed in the HSU+PEMF group than in the HSU group. These results confirmed that PEMFs with proper parameters can promote the healing of injured tissues under hypoxia, especially in the organs, which are nearly similar to our previous research results on the influence of PEMFs on plateau frostbite [16]. However, the effect of PEMFs on inflammatory cells was verified during the detection of TNF-*α* protein, which is closely associated with inflammation indexes. PEMFs obviously promoted the decrease in TNF-*α* protein on the basis of the results of TNF-*α* protein measurement. Therefore, the promotion may accelerate HSU healing. The combined effects of PEMFs were positive during HSU healing.

In this study, the more ideal results of the effects of PEMF on HSU should be obtained in a continuous or periodical hypoxic environment. However, we found that the extremely difficult conditions have made some rats too ill to survive for other complications, such as cerebral edema. Therefore, we checked the effects of PEMF on HSU in the normal environment. When other complications could be controlled, investigating the effects of PEMF on HSU with continuous hypoxic would be truly significant; thus a perfect design should be performed in the future.

## 5. Conclusions

Our study demonstrated that PEMFs can accelerate HSU restoration, which is evidenced by quantitative gastric juice pH value and UI observation, histological results, TNF-*α* protein, and NE detection. Based on the results, the findings indicated that PEMF, as an effective noninvasive and accessible therapeutic method, might provide an exciting therapeutic alternative for HSU treatment.

Under some special conditions, PEMF, as an adjuvant therapy, could be combined with drugs to treat gastric ulcer, the actual effects of which require subsequent experiments.

## Figures and Tables

**Table 1:** Comparison of NE concentrations (ng/g) between each group on Days 1, 3, 5, and 7.

Group	NE concentrations (ng/g)			
	Day 1	Day 3	Day 5	Day 7
Control	182.81±11.20	183.32±4.52	182.19±9.56	182.33±6.17
HSU	276.25±7.12 ^b^	268.54±6.35 ^b^	255.55±7.36 ^b^	249.36±4.36 ^b^
HSU+PEMF	197.23±9.21^b,c^	195.71±7.12 ^b,c ^	192±6.32 ^b,c^	186.23±9.29 ^b,c^

Values are expressed as mean ± SD.

^b^ *P* < 0.01 versus control group.

^c^ *P *< 0.05 versus HSU group.

## Data Availability

The detailed data of Figures 2 and 4 used to support the findings of this study are available from the corresponding author upon request. The other data used to support the findings of this study are included within the article.
